# Progressive Multifocal Leukoencephalopathy

**DOI:** 10.12688/f1000research.7071.1

**Published:** 2015-12-10

**Authors:** Laura Adang, Joseph Berger

**Affiliations:** 1 Division of Neurology, Children's Hospital of Philadelphia, Philadelphia, Pennsylvania, 19104, USA; 2 Department of Neurology, Hospital of the University of Pennsylvania, Philadelphia, Pennsylvania, 19104, USA

**Keywords:** Progressive multifocal leukoencephalopathy, demyelination, JC virus, immunocompromised, highly active antiretroviral therapy

## Abstract

Progressive multifocal leukoencephalopathy (PML) is a devastating demyelinating disease with significant morbidity and mortality and no effective, targeted therapies. It is most often observed in association with abnormalities of cell-mediated immunity, in particular human immunodeficiency virus (HIV) infection, but also occurs in association with lymphoproliferative diseases, certain immunosuppressive and immunomodulatory regimens, and other conditions. The etiologic agent of PML is a small, ubiquitous polyomavirus, the JC virus (JCV, also known as JCPyV), for which at least 50% of the adult general population is seropositive. PML results when JCV replicates within cerebral oligodendrocytes and astrocytes, leading to oligodendrocyte death and demyelination. Unfortunately, no treatments have been convincingly demonstrated to be effective, though some have been employed in desperation; treatment otherwise includes attempts to restore any immune system defect, such as the withdrawal of the causative agent if possible, and general supportive care.

## Introduction

Progressive multifocal leukoencephalopathy (PML), a typically rapidly progressive, potentially fatal neurologic syndrome, was first described in 1958 as a complication of chronic lymphocytic leukemia and Hodgkin’s disease ^1^. Within two decades of its initial description, in the 1970s, the JC virus (JCV, also known as JCPyV) was discovered as the etiologic agent ^2^. It is named for a subject with PML − patient John Cunningham ^3^. Due to its rarity (1.3 cases per 1000 person-years at risk in human immunodeficiency virus [HIV]+ patients), the disorder is regarded as an orphan disease ^4^. However, following the acquired immunodeficiency syndrome (AIDS) pandemic and with newer immunomodulatory therapies, such as natalizumab, that predispose to the development of PML, the incidence of the disease has increased substantially.

## The JC Virus

A ubiquitous infection, the JCV infects over half of the adult population globally ^3, 5, 6^. Typically the initial exposure occurs during childhood. As no identified clinical illness accompanies acute infection, it is believed that initial infection results in a transient asymptomatic viremia following which the virus establishes as a latent or persistent infection in the kidney and perhaps elsewhere in the body ^3^. The JCPyV found in the urine of approximately one-third of all adults is referred to as the archetype virus and is incapable of replicating effectively in glial tissue. It is theorized that the archetypal form of the virus is responsible for the primary infection. The mode of transmission is also unclear, although it is suspected to be through the tonsils or gastrointestinal tract ^3^. Interestingly, even among the immunocompromised, only a small subset of infected patients develop PML, as the development of this syndrome requires a complex series of events: the virus must be transformed to the prototype (neurotropic) virus, seed the brain, and avoid neuro-immunosurveillance and clearance. Generally, this occurs in the setting of an impairment in cell-mediated immunity, as with HIV/AIDS, or with the use of immunomodulatory agents, such as natalizumab. The transformation of JCV from the archetype to the prototype virus requires genetic modifications in the non-coding control region of the viral DNA. This transformation of the small, circular JCPyV DNA genome impacts the replicative ability, gene transcription patterns, and homing within the body, and ultimately disease pathology ^3^.

## PML and the JC virus

In immunocompetent individuals, the JCV is rarely pathogenic, but in immunocompromised patients, it may cause PML, an aggressive, progressive neurologic syndrome that is potentially devastating. Prior to the availability of highly active antiretroviral therapy (HAART), PML was observed in 5–10% of all persons with AIDS, and HIV/AIDS has been an underlying predisposing cause of PML in more than one-half of individuals ^3, 7^. Following the advent of HAART, the incidence of PML in this population has declined ^6^. Another cluster of PML cases is observed in patients receiving immunomodulatory therapies. Two therapies in particular appear to predispose to PML, namely natalizumab (trade name Tysabri) and efalizumab (now off the market) ^8^. However, PML has been reported with the use of rituximab, belatacept, fingolimod, infliximab, alemtuzumab, mycophenolate mofetil, fludarabine, leflunomide, and fumaric acid esters as well ^9^. The increased risk of PML from natalizumab is thought to be due to the known mechanism of the drug, namely α4β1 integrin binding. In so doing, this monoclonal antibody prevents lymphocytes from binding to vascular cell adhesion molecule 1 (VCAM) on the central nervous system (CNS) endothelium, decreasing CNS immune surveillance ^10^. While an immune-modulated state is relatively common, PML remains a rare disorder even within these subpopulations. This suggests that immunosuppression alone is insufficient to reactivate the JCV and cause disease.

While the JCV predominantly results in the clinical syndrome of PML, it can also cause granule cell neuronopathy (GCN), JCV encephalopathy, and even isolated JCV meningitis ^11^. In GCN, the granule cell neurons of the cerebellum are affected, resulting in symptoms including ataxia, tremor, and nystagmus ^12^. JCV encephalopathy is pathologically characterized by the infection and lysis of the cortical gray matter ^13^.

## Diagnosis of PML

The diagnosis of PML requires clinical, radiographic, and virologic evidence ^5^. Clinically, PML can present with a wide constellation of neurologic signs and symptoms due to its ability to affect virtually any area of the brain and the frequently multifocal nature of the lesions. In patients with multiple sclerosis (MS) on natalizumab or other disease-modifying drugs that seem to predispose to PML, distinguishing PML from an acute MS attack can be difficult, as the general symptoms can be similar to the symptoms of an MS flare. The most commonly reported symptoms include gait changes, weakness, cognitive impairment, sensory symptoms, headache, and visual changes ^14^. Visual symptoms are reported in one-quarter to one-half of all PML patients, typically presenting as a field deficit, and can be the initial symptom as well ^15^. Visual system involvement is secondary to involvement of the visual pathways and not as a direct optic neuritis, as seen in other inflammatory, demyelinating diseases ^15^.

Seizures can occur in up to one-third of the general PML population and are more frequent with juxtacortical and T1-hyperintense lesions by magnetic resonance imaging (MRI) ^13^. There is subtle variation in the presentation of PML as determined by the underlying cause: HIV-associated PML vs. immunosuppressant-associated PML. Unlike PML associated with HIV infection where 50% have predominant motor system findings ^7^, natalizumab-associated PML appears to be more commonly associated with cognitive and language changes; motor symptoms are reported in only one-third of natalizumab PML patients ^14^.

Pathologically, PML is characterized by multifocal demyelination, with smaller lesions coalescing into larger foci. Overall lesion burden can be extensive, involving entire hemispheres, and can occur throughout the white matter. By histopathology, PML is characterized by multifocal demyelination, enlarged astrocytes that contain lobulated hyperchromatic nuclei and oligodendrocytic hyperpigmented enlarged nuclei ^16^. JC virions can be found by electron microscopy, particularly within reactive astrocytes.

The appearance of PML by cerebral imaging can be heterogeneous, although it is typically multifocal with frontal or parieto-occipital locations predominating. There are rare reports of PML involvement isolated to the deep gray matter or the brainstem, or even resulting in spinal cord parenchyma abnormalities, but, to date, clinical features of a myelopathy have not been reported with PML ^17^. On computed tomography (CT) imaging, PML lesions are hypointense within the white matter. When the subcortical arcuate fibers are involved, the lesions can have a ‘scalloped’ appearance. By MRI, a far more sensitive measure to detect evidence of PML, lesions are hyperintense by T2 and fluid-attenuated inversion recovery (FLAIR) imaging and hypointense by T1. Gadolinium enhancement is more common in natalizumab cases, one-third vs. 15% of HIV-associated cases ^14^. Of note, similar radiographic patterns can also be found in cytomegalovirus infections, acute disseminated encephalomyelitis, varicella-zoster leukoencephalopathy, and brain tumors, although the diagnosis is often evident within the context of a full history and physical examination ^14^. Patients prescribed medications such as natalizumab should be annually screened by MRI for the imaging characteristics of PML, as it is possible to detect the radiographic findings before the clinical onset.

To develop PML, a patient must have a latent or persistent infection with the JCV. This is believed to be in an extraneural reservoir, chiefly the kidney. The virus then undergoes genetic rearrangement into the neurotropic form, infects oligodendrocytes and astrocytes within the CNS, and leads to demyelination from oligodendrocyte lysis when the host cannot mount an appropriate immune response to contain the virus. The value of cerebrospinal fluid (CSF) polymerase chain reaction (PCR) for JCPyV is highly dependent on the nature of the test; ultrasensitive PCR techniques for JCPyV have a sensitivity of >95%. Patients on natalizumab at risk for the development of PML are typically screened for the presence of JCV-specific antibodies. A semi-quantitative antibody index is used to indirectly track JC infection and prognosticate PML risk. JCV antibody index exceeding 1.5 has been associated with a higher risk of PML ^18^. Other factors significantly increasing the risk for natalizumab-associated PML is prior immunosuppressive therapy use and duration of natalizumab (>24 months) therapy ^19^.

## PML survival and treatment

PML is an aggressive, potentially fatal disease. In individuals in whom the immune dysfunction can be restored, such as patients with AIDS in whom immune function is restored by antiretroviral therapy, survival is improved ^20^. Presumably, the same is true of patients on immunosuppressive agents, such as natalizumab, who are diagnosed early and treated with drug cessation, plasma exchange to more quickly eliminate the natalizumab, and supportive care ^4, 14, 21^. Mortality in natalizumab-related cases is approximately 21% ^22, 23^. Survival in the natalizumab cases was associated with younger age, lower pre-PML functional disability, lower viral loads, and more focal brain involvement ^23^. Survival in HIV-associated PML cases is dependent on CD4 count and ranges from 50% to 80% ^21^. PML may be associated with the immune reconstitution syndrome (IRIS) with recovery of immune function. IRIS is defined as a worsening of radiographic and clinical findings of an infection in the context of immune system recovery. Accordingly, patients are often treated with corticosteroids concurrent with supportive care and plasma exchange, although the benefit of this approach still remains unproven ^9^. Among natalizumab-associated PML survivors, one-third of the patients have mild neurologic deficits, one-third have moderate impairment, and one-third are severely affected ^22, 24^. Despite the efficacy of certain therapeutic agents in decreasing JCPyV replication *in vitro*, such as mefloquine and cidofovir, none have demonstrated efficacy in randomized controlled trials.

In conclusion, PML is an aggressive brain infection caused by the JCV, almost exclusively found in immunosuppressed patients. Although consensus is that high-risk patients on immunosuppressant medications such as natalizumab should be monitored by serial imaging and anti-JCV antibody screening, the frequency of testing and the threshold for concern is a rapidly moving target. The mainstays of treatment include stopping the inciting agent and plasma exchange, although directed anti-viral therapeutics is an active area of investigation. While the duration of therapy with natalizumab influences the risk, many other factors are involved in the development of PML, suggesting a complexity to the predisposition to PML development.

## Abbreviations

AIDS - Acquired immunodeficiency syndrome

PML - Progressive multifocal leukoencephalopathy

HIV - Human immunodeficiency virus

CNS - Central nervous system

CT - Computed tomography

IRIS - Immune reconstitution syndrome

JC - John Cunningham

JCV - John Cunningham virus

JCPyV - John Cunningham polyomavirus

MRI - Magnetic resonance imaging

HAART - Highly active antiretroviral therapy

VCAM - Vascular cell adhesion molecule 1

GCN - Granule cell neuronopathy

MS - Multiple sclerosis 

CSF - Cerebrospinal fluid

PCR - Polymerase chain reaction
